# New *Pseudomonas* infections drive Pf phage transmission in CF airways

**DOI:** 10.1172/jci.insight.188146

**Published:** 2025-04-22

**Authors:** Julie D. Pourtois, Naomi L. Haddock, Aditi Gupta, Arya Khosravi, Hunter A. Martinez, Amelia K. Schmidt, Prema S. Prakash, Ronit Jain, Piper Fleming, Tony H. Chang, Carlos Milla, Patrick R. Secor, Giulio A. De Leo, Paul L. Bollyky, Elizabeth B. Burgener

**Affiliations:** 1 Biology Department, Stanford University, Stanford, California, USA.; 2 Division of Infectious Diseases and Geographic Medicine, Department of Medicine, Stanford University School of Medicine, Stanford, California, USA.; 3 Division of Biological Sciences, University of Montana, Missoula, Montana, USA.; 4 Oceans Department, Stanford University, Pacific Grove, California, USA.; 5 Center for Excellence in Pulmonary Biology, Division of Pulmonary Medicine, Department of Pediatrics, Stanford University, Stanford, California, USA.; 6 Department of Microbiology and Cell Biology, Montana State University, Bozeman, Montana, USA.; 7 Division of Pediatric Pulmonology & Sleep Medicine, Department of Pediatrics, Children’s Hospital Los Angeles, Keck School of Medicine at University of Southern California, Los Angeles, California, USA.

**Keywords:** Infectious disease, Microbiology, Bacterial infections, Fibrosis

## Abstract

Pf bacteriophages, lysogenic viruses that infect *Pseudomonas aeruginosa* (*Pa*), are implicated in the pathogenesis of chronic *Pa* infections; phage-infected (Pf^+^) strains are known to predominate in people with cystic fibrosis (pwCF) who are older and have more severe disease. However, the transmission patterns of Pf underlying the progressive dominance of Pf^+^ strains are unclear. In particular, it is unknown whether phage transmission commonly occurs horizontally between bacteria via viral particles within the airway or whether Pf^+^ bacteria are mostly acquired via de novo *Pseudomonas* infections. Here, we studied *Pa* genomic sequences from 3 patient cohorts totaling 662 clinical isolates from 105 pwCF. We identified Pf^+^ isolates and analyzed transmission patterns of Pf within patients between genetically similar groups of bacteria called “clone types.” We found that Pf was predominantly passed down vertically within *Pa* clone types and rarely via horizontal transfer between clone types within the airway. Conversely, we found extensive evidence of *Pa* de novo infection by a new, genetically distinct Pf^+^ *Pa*. Finally, we observed that clinical isolates showed reduced activity of type IV pili and reduced susceptibility to Pf in vitro. These results cast light on the transmission of virulence-associated phages in the clinical setting.

## Abstract 2

Pf bacteriophages infecting P. aeruginosa in people with cystic fibrosis are passed down within lineages but are rarely transmitted in patients’ airway.

## Introduction

Bacteriophages, viruses that parasitize bacteria, have complex relationships with their bacterial hosts. Purely lytic phages always lyse their hosts and can help clear bacterial infections (1, 2). Conversely, lysogenic phages integrate their genetic material into the bacterial genome and reproduce via bacterial replication (i.e., lysogeny) or through opportunistic lysis (3, 4). Finally, some phages can also produce new viral particles without killing the cell, resulting in chronic infections of the bacterial cell (5–7). These phages can have a much more nuanced effect on bacterial populations, sometimes promoting treatment failure (4, 8–10). One lysogenic phage that has been implicated in the pathogenic behavior of its bacterial host is the filamentous phage Pf, which is harbored by *Pseudomonas aeruginosa* (*Pa*) (9–11).

Pf phages are inoviruses with a single-stranded DNA genome encased in a filamentous structure a few nanometers wide and 1–2 μm long (5, 6). They include Pf1 to Pf8 phages, in addition to other yet unassigned Pf phages, and are prevalent in multiple well-described lab strains of *Pa* (6, 9, 12, 13). Filamentous phages like Pf are temperate and therefore have 2 possible routes of transmission. They have the ability to both integrate their 6000- to 15,000-bp genome into the genome of their bacterial host (vertical transmission) and to produce new viral particles that infect susceptible bacterial hosts by binding to the type IV pili (horizontal transmission) (14–16). Filamentous phages form a non-lytic chronic infection during which progeny phages are produced and extruded without killing the bacterial host, instead of release by lysis as is the case with lytic phages (5, 17).

The bacterial host of Pf phage, *Pa*, is a Gram-negative bacterium that is a common pathogen of humans. *Pa* is often resistant to multiple antibiotics, leading to its recognition as a pathogen of concern by the World Health Organization (18). It is present in the environment and is responsible for opportunistic infections of burns and diabetic wounds as well as infections in immunocompromised individuals (19–21). In particular, *Pa* is one of the most common bacteria found in lung infections in people with cystic fibrosis (pwCF) (22).

*Pa* is particularly problematic in CF, a hereditary disease characterized by the disruption of ion channels (23). Symptoms include the accumulation of thickened mucus in the airways, leading to chronic bacterial infections and reduced lung function leading to eventual respiratory failure (24). Infection with *Pa* is a major predictor of morbidity and mortality in pwCF (25, 26).

Worse outcomes in pwCF are associated with Pf presence (9, 10, 27), and phage-infected (Pf^+^) *Pa* strains are known to predominate in older, more chronically ill pwCF. Similar findings have been reported in patients with chronic wounds infected with *Pa*, where Pf can be detected in the wounds, and more so in larger, slower-to-heal wounds (28). This dominance is thought to reflect selective advantages conferred by Pf through the modulation of phagocytosis (29–32) and the physical interaction of viral particles and biofilm polymers producing a crystalline organization of the biofilm, which reduces the efficacy of antibiotic treatment and thereby promotes antibiotic tolerance (33–36). Specifically, a charged-based interaction between negatively charged Pf phages and positively charged antibiotics results in the sequestration of these antibiotics away from bacteria and increased bacterial survival during antibiotic treatment (35, 37). In addition, genes encoded by Pf result in modified quorum sensing signaling, inhibition of pyocyanin production, and protection against superinfection by other Pf phages through suppression of the type IV pili (38–40).

Overall, Pf phages contribute to the fitness and pathogenicity of *Pa*, especially under antibiotic treatment, and favor the development of chronic infections (10, 11, 36). In pwCF, this manifests as Pf^+^ infections being associated with advanced disease and more severe pulmonary exacerbations (9). There are also reports that Pf phage is associated with bacterial phenotypes linked to reduced virulence and chronic infection (41–43). Yet, little is known about the transmission of Pf phages in the clinical setting, which involves heterogeneous environments (44–46) and multiple bacterial clone types — closely related isolates — both within and between patients (47–52).

The airways of pwCF present a unique environment, with bacteria and phages navigating a highly complex spatial structure (44–46, 53). In addition, *Pa* has been shown to adapt to the CF airway and exhibit changes in metabolism, antibiotic resistance, motility, and other virulence factors that could affect Pf infection (46, 48, 54). While the prevalence of Pf-infected bacteria increases with patients’ age (9), the mechanisms driving this increase are unclear. In particular, we do not know whether Pf^+^ strains are generally acquired directly from the environment and become more prevalent in a patient with time as a result of the competitive advantage of Pf^+^ bacteria, especially under antibiotic treatment, or whether horizontal transmission of phages among bacteria within a patient’s airway commonly contributes to this increase.

In this study, we focused on Pf phages of *Pa* infecting the airways of pwCF. We used bacterial genomes from 3 different patient cohorts to describe patterns of infection by Pf and investigate how Pf phages spread within patients. We used genomic data from bacterial samples from 2 different cohorts of pwCF already published in the literature (48, 49) and newly sequenced samples from a third cohort of patients from the Cystic Fibrosis Center at Stanford to investigate patterns in Pf transmission in *Pa* airway infections in pwCF. We asked whether Pf phages are often transmitted horizontally within patients or whether Pf^+^ infections usually represent new clone types in the airway infection. We observed a single instance of a Pf^–^ clone type being subsequently infected by a Pf phage in the airway, out of the 102 clone types in this study. In addition, we observed that all clinical isolates tested showed reduced type IV pilus function and susceptibility to Pf. These results suggest that new Pf^+^ infections are most commonly caused by a new bacterial infection with a genetically distinct Pf^+^ *Pa* clone rather than horizontal transmission of Pf from one clone to another within an established *Pa* infection.

## Results

**Figure 1:**
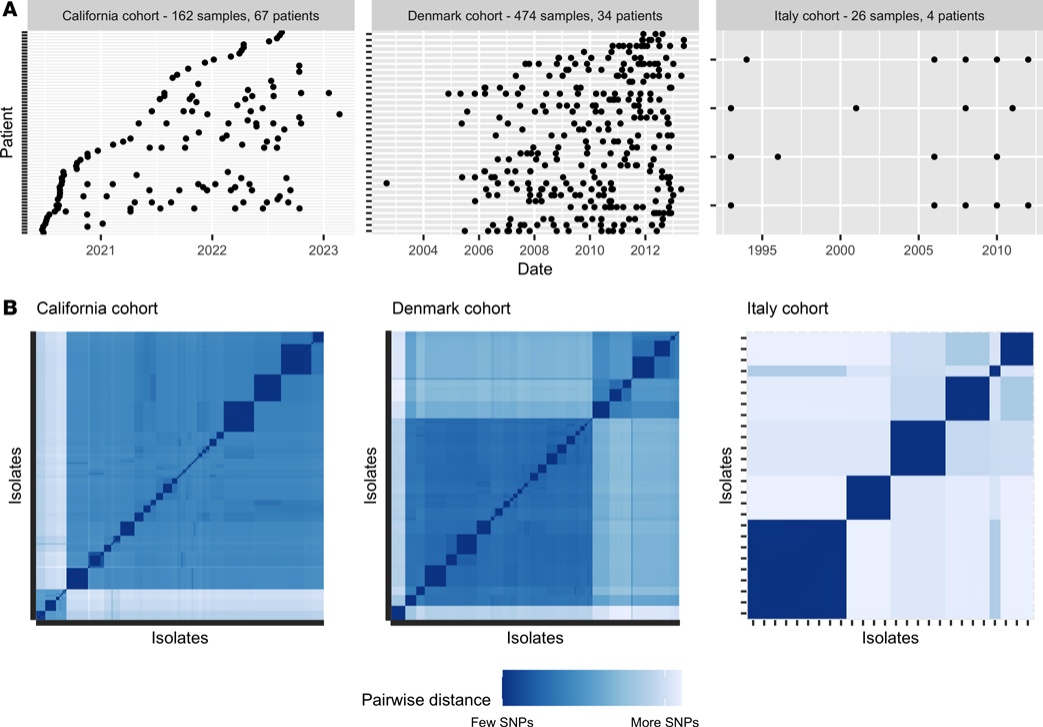
Overview of the 3 patient cohorts used in this study. (**A**) Time series of sample collection from patients for each cohort. (**B**) Genetic distance matrix showing pairwise SNPs between each isolate for each cohort. All isolates were clustered using SNPs so that more similar isolates are located closer to each other on the *x* and *y* scales. The diagonal represents the comparison of each isolate to itself (0 SNPs). Clinical isolates cluster into groups of high genetic similarity (small dark blue squares) called clone types. In addition, larger clusters (medium blue squares) are observed. Genetic distance scale varies between cohorts.

We collected and sequenced bacterial isolates from pwCF treated at the Cystic Fibrosis Center at Stanford (California cohort). In total, 162 bacterial samples were sequenced, across 67 pwCF over nearly 3 years (Figure 1A). We combined these with publicly available sequences from a cohort of Danish and a cohort of Italian pwCF. These datasets contain 474 and 26 bacterial isolates from 34 and 4 pwCF, across 11 and 19 years, respectively (Figure 1A) (48, 49). Isolates from each cohort clustered into groups of high genetic similarity that have recently diverged within individuals and are characterized by fewer than 10,000 SNPs (Figure 1B). Following Marvig et al. (48), we refer to these groups as clone types. If a new clone type appears in a patient, we consider that a de novo *Pa* infection. We validated that different clone types within a patient did not diverge from each other and represented de novo *Pa* infections using phylogenetic relationships (Supplemental Figure 1; supplemental material available online with this article; https://doi.org/10.1172/jci.insight.188146DS1).

### Pf phages were found in high proportions across the 3 patient cohorts.

We first asked how prevalent Pf phages are across patient cohorts in different countries. Lineages of Pf phages target distinct integration sites to integrate themselves into bacterial genomes (13). A single bacterium can thus be infected by multiple Pf phages (55).

**Figure 2:**
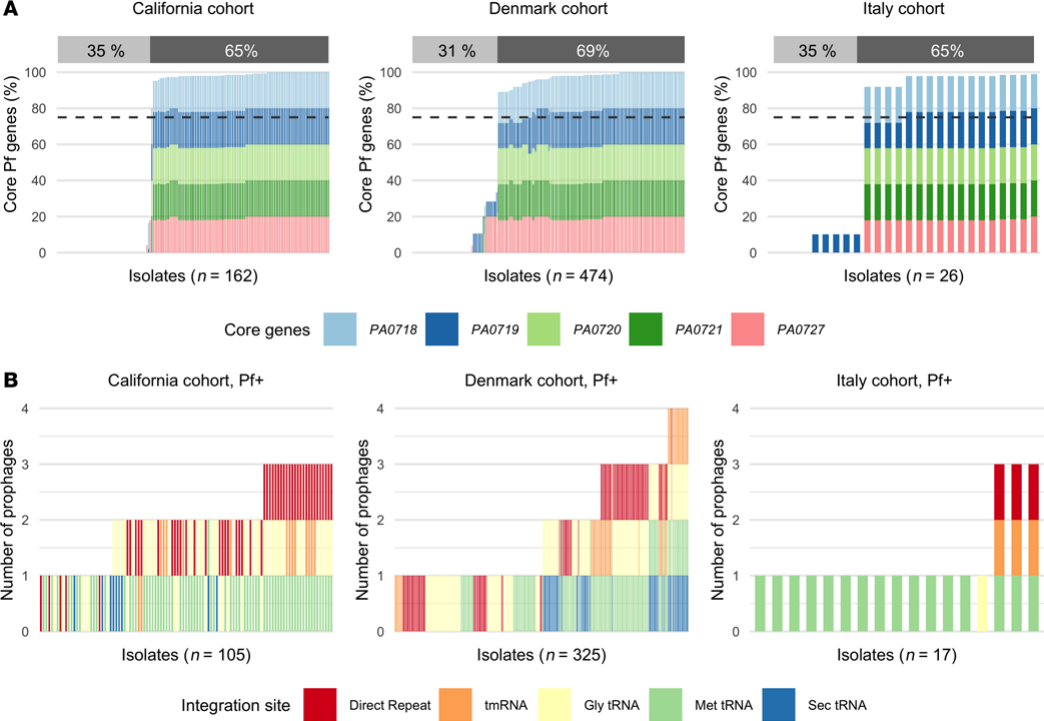
Majority of *Pa* infected by Pf phages. (**A**) Percentage of core Pf genes *PA0718*, *PA0719*, *PA0720*, *PA0721*, and *PA0727* found in isolates for each patient cohort. The percentage of Pf^+^ isolates, defined with a minimum average coverage of 75% for these 5 core genes, is shown above each graph. (**B**) Number of prophages found in Pf^+^ isolates, as defined above, and integration site used by these Pf phages for each patient cohort. Integration sites are sequences in the bacterial genomes recognized by lysogenic phages to integrate into the bacterial chromosome. Different Pf lineages are able to use different integration sites, based on the presence of the corresponding integrase gene in the Pf genome.

We used the presence of 5 highly conserved Pf genes — *PA0718*, *PA0719*, *PA0720*, *PA0721*, and *PA0727* — to detect Pf prophages in the chromosome of *Pa* clinical isolates (13). Using a threshold of 75% total coverage of these core genes, we found that between 65% and 69% of isolates from each *Pa* patient cohort were Pf^+^ (Figure 2A). These results were not sensitive to changes in coverage threshold between 35% and 85% (Supplemental Figure 2).

The number of Pf prophages infecting each isolate was then determined using variation in the Pf integrase gene *PA0728*, also called *intF* (Figure 2B). Each integration site in the *Pa* chromosome can be targeted by different Pf phages with the corresponding integrase. We thus used the 5 different integrases described in the literature (see Methods) to categorize Pf phages into 5 different Pf “types” and to count the number of phages infecting each isolate. We validated this approach using long-read sequencing of 12 isolates with different predicted numbers of phages and did not find any instances of multiple infections by phages with the same integration site in a single bacterial isolate. Coinfection occurred in many isolates, with up to 4 different Pf prophages per isolate in the cohort from Denmark (Figure 2B). The most common integration sites used by Pf phages were Met-tRNA and Gly-tRNA (32% and 28%, respectively), followed by direct repeats (21%). These integration sites are used by well-described Pf phages, with reference Pf phages Pf4, Pf5, Pf6, and Pf7 using Gly-tRNA, direct repeats, Met-tRNA, and Met-tRNA, respectively (13). The least commonly found integration sites were tmRNA and Sec-tRNA at 10% and 9%, respectively.

### Gain or loss of Pf phage by clone types is rare during chronic infections of pwCF.

**Figure 3:**
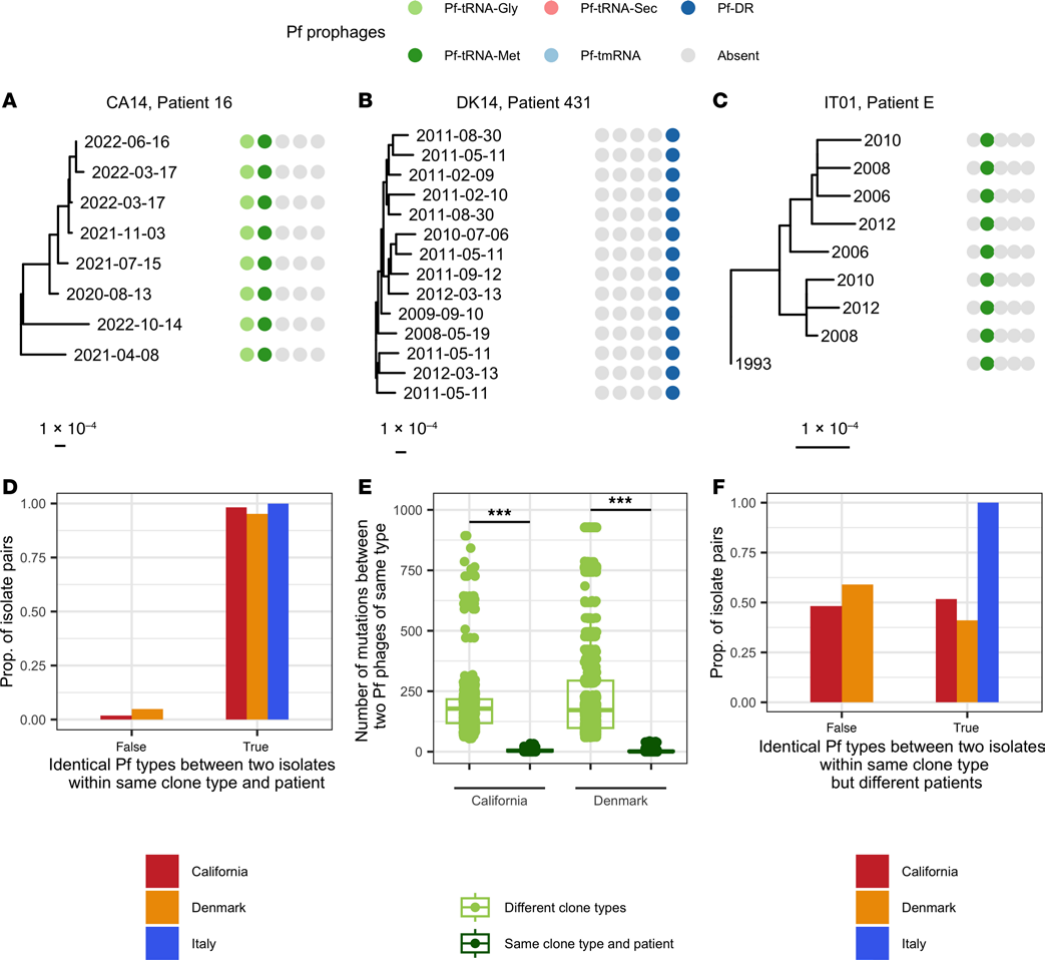
Pf prophages are maintained within clone types within patients. Presence or absence of 5 different Pf types, as defined by their integrase, for 1 clone type and patient in the California cohort (**A**), Denmark cohort (**B**), and Italy cohort (**C**). Sample dates are shown at the end of each branch. (**D**) Proportion of *Pa* isolate pairs with identical Pf infection patterns (same number and type of Pf phages, as determined by their integrase) for isolates of the same clone type and in the same patient. (**E**) Number of non-identical nucleotides (including SNPs and large insertions/deletions) for pairwise comparisons of Pf genomes of the same phage type (using the same integrase) for Pf infecting the same patient and *Pa* clone type, and for Pf infecting different clone types. Pf phages were more likely to share more of their genome if they infected the same clone type in the same patient. ***P < 0.001 by Wilcoxon’s rank-sum test. Boxes show the median (horizontal lines) and interquartile range (bounds of the boxes) and all data points are shown. (**F**) Proportion of *Pa* isolate pairs of the same clone type but in different patients with identical Pf infection patterns (same number and type of Pf phages, as determined by their integrase).

We mapped the presence of Pf phages with different integrases onto phylogenetic trees of the bacterial isolates for each cohort. Pf^+^ and Pf^–^ bacteria were present across the phylogenetic tree, while the number of Pf phages was usually conserved within clone types isolated from the same patient (e.g., clone types CA14, DK14, and IT01; Figure 3, A–C). Specifically, 98%, 95%, and 100% of isolate pairs from the same clone type in the same patient were infected by the same number and same type of phages, as defined by their integrase, for the California, Denmark, and Italy patient cohorts, respectively (Figure 3D). Isolates of the same clone type isolated from a single patient were significantly more likely to be infected by the same Pf phages than other isolate pairs for all clinical cohorts (χ^2^ test, *P* < 0.001 for all). Furthermore, Pf phages of the same type, as determined by their integrases, infecting bacteria of the same clone type within the same patient had a significantly lower number of non-identical nucleotides (whether from SNPs or indels) than Pf phages of the same type infecting different clone types or different patients for both the California and Denmark cohorts (Wilcoxon’s rank-sum test, *P* < 0.001 for both; Figure 3E). In other words, Pf phages of the same type in the same patient were likely to have diverged within that patient, rather than to represent a new Pf phage from the same type acquired from the environment or other patients. Together, these results suggest that clone types do not typically gain new phages, whether of the same type or not, at the timescale of divergence of clone types over multiple years in the airways. Note that the low number of mutations observed in Pf phages due to their small genome size (~10,000 bp) did not allow us to evaluate Pf transmission of a particular phage type within a bacterial clone type.

We then asked whether Pf transmission was occurring more generally, either in other infections or in the environment. We observed that 53%, 41%, and 100% of isolate pairs from the same clone type but isolated from different patients were infected by the same Pf phages in the California, Denmark, and Italy cohorts, respectively (Figure 3F). The proportion of isolate pairs that had the same number and same type of phages was significantly lower for isolates of the same clone types isolated from different patients than the pairs isolated from the same patients for the California and Denmark cohorts (Figure 3, D and F; χ^2^ test, *P* < 0.001 for both). There were no isolates of the same clone type in different patients for the Italy cohort. These data suggest that Pf transmission is common in the environment or in other types of *Pa* infections but may be prevented during CF airway infections by the phenotypes of *Pa* that have adapted to the CF airway.

### De novo infection by a new Pa clone type, rather than horizontal transmission, is responsible for most new Pf^+^ infections in pwCF.

**Figure 4:**
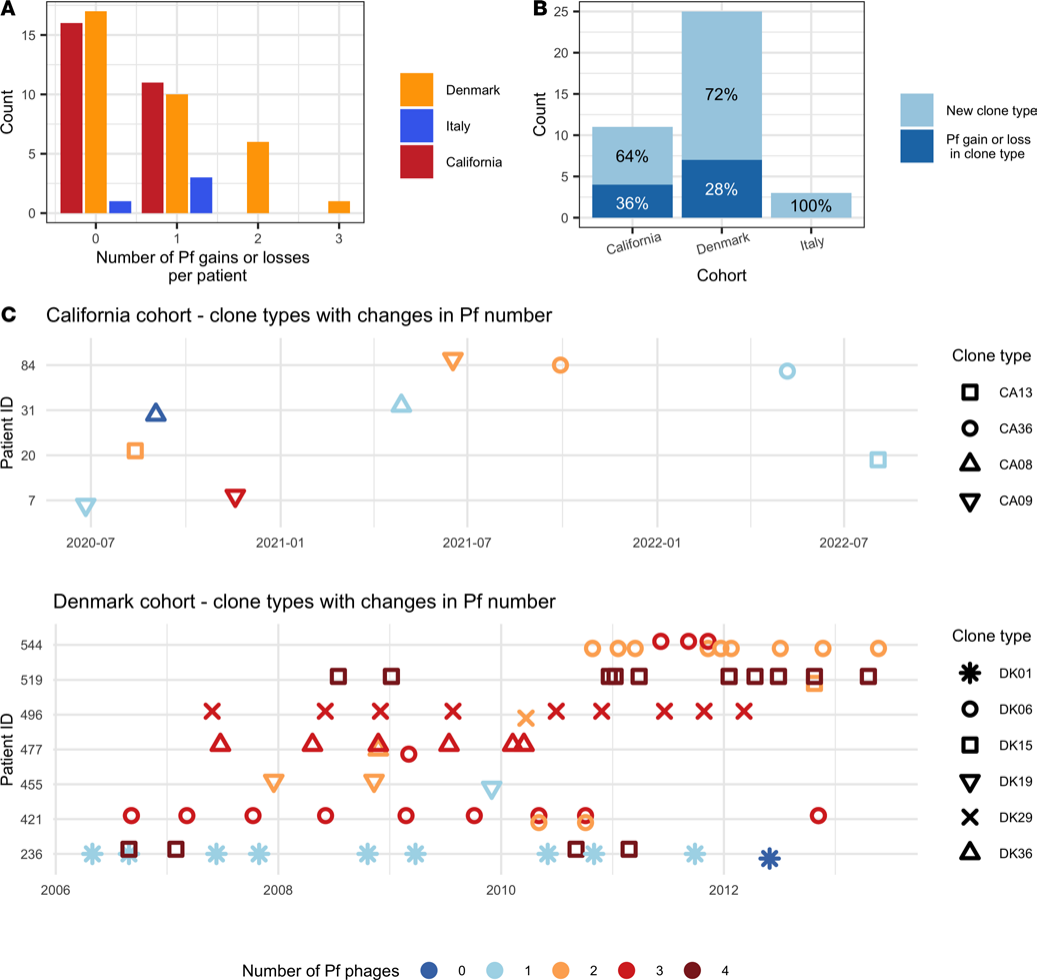
The majority of changes in the number of Pf phages in patients are associated with colonization or dominance of new clone types. (**A**) Number of changes in the number of Pf phages per patient. (**B**) Percentage of the changes in the number of Pf phages that are due to a change in clone type or to infection of a clone type by Pf (horizontal transmission). (**C**) Time series of the number of phages and clone types for patients with at least one change in the number of phages in a clone type over time for the California cohort and for the Denmark cohort. No Pf change occurred within a clone type for any patient of the Italian cohort. Different shapes represent different clone types, while color indicates the number of Pf prophages found in that isolate.

Many patients carried either only Pf^–^ or Pf^+^ isolates over the study period, with 60%, 50%, and 25% of patients with multiple samples seeing no change in the number of Pf prophages for the California, Denmark, and Italy cohorts, respectively (Figure 4A). When patients were infected with isolates with different numbers of phages, 64%, 72%, and 100% of the changes in Pf prophage copy numbers were associated with a change in clone type for the California, Denmark, and Italy cohorts, respectively (Figure 4B). All changes from Pf^–^ to Pf^+^ (independently of prophage number) represented a change in clone type in all cohorts, except for clone type CA08 in patient 31 in the California cohort (Figure 4C). Most of the other clone types (CA36, CA13, DK01, DK15, DK19, DK29, and DK36) lost phages over time, often transiently (likely representing a transient change in the dominant strain), while CA09 gained multiple Pf phages and DK06 showed both gains and losses in different patients. Pf^+^ infections in patients were thus usually caused by infection by a new clone type, rather than by the gain of a new Pf prophage by an existing Pf^–^ clone type, as Pf phage loss accounted for most changes in Pf numbers observed in the 36% and 28% of clone types that went through a change in Pf numbers in patients.

Some *Pa* clone types infecting pwCF accumulate mutations in the DNA mismatch repair genes *mutS* and *mutL*, resulting in hypermutator phenotypes. This could affect Pf transmission, either through increasing mutations of Pf receptor genes, which would decrease horizontal transmission, or increasing mutations of the Pf prophage itself. Mutations in Pf phages can result in superinfective Pf phages (42), which are better at infecting and lysing Pf^+^ *Pa*, and would thus increase Pf transmission to other clone types. We thus asked whether the clone types that gained Pf phages over time (CA08, CA09, and DK06), and any clone types found infecting the same patients, had mutations in *mutS* or *mutL*. Clone type DK06 and DK37 infected patient 421. Neither DK06 nor DK37 was found to have accumulated non-synonymous mutations in mismatch repair genes. In the California cohort, we found new mutations in *mutS* or *mutL* for 7 clone types, including CA08, which gained a phage in patient 31. Five out of these 7 clone types gained or lost Pf phages, whether within or between patients. Out of 2 coinfecting clone types of CA08, one had mutations in *mutL*. We did not find mutations for CA09, but we did find *mutS* and *mutL* mutations in 2 out of 5 of its coinfecting clone types (*mutL* mutations for CA37 in patient 84 and *mutS* mutations for CA13 in patient 97). The low number of clone types gaining or losing Pf phages did not allow us to quantitatively assess the effect of hypermutator phenotypes on Pf transmission.

Finally, we observed a dichotomy in clone type persistence between patients. Patients with changes in clone types and Pf status often went through many of these changes over time (Figure 4C), while other patients remained infected by the same clone type for decades (Figure 4A and Supplemental Figure 3). Time series for patients without a change in Pf and with a change in Pf due to superinfection by new clone types are available in Supplemental Figures 3 and 4, respectively.

### Clinical isolates do not twitch and show decreased susceptibility to infection by Pf phages in vitro.

**Figure 5:**
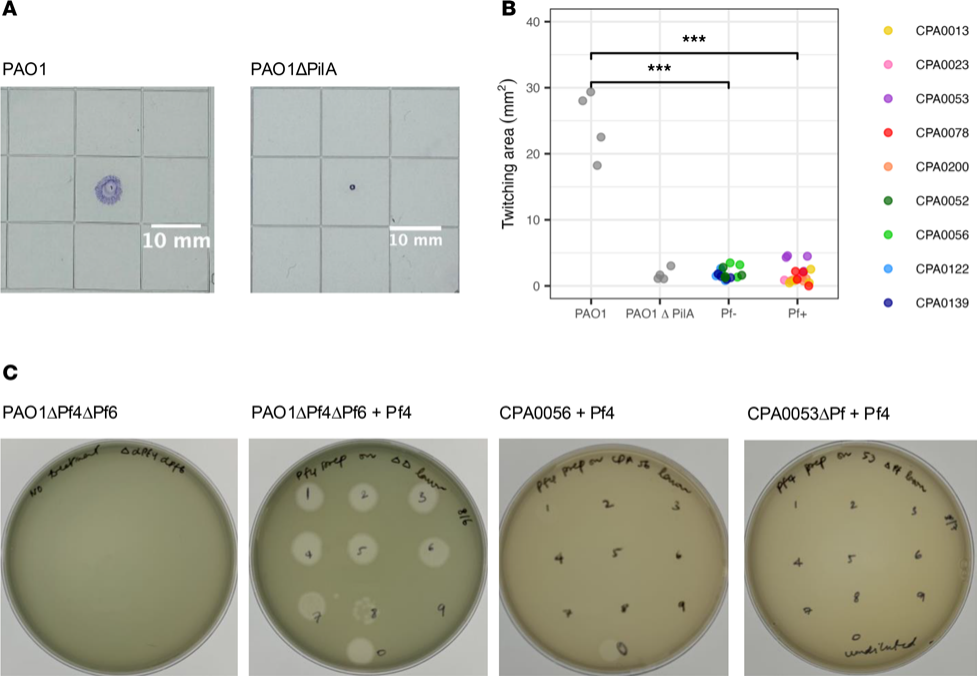
Clinical isolates do not twitch and are less susceptible to Pf than PAO1. Clinical isolates were selected from patients who were infected by both Pf^–^ and Pf^+^ isolates and that showed sufficient growth in vitro. (**A**) Positive (PAO1) and negative (PAO1ΔPilA) controls for twitching assays, showing motility of bacteria over agar after 24 hours. PAO1ΔPilA mutants do not have functional type IV pili and cannot twitch, resulting in a small stained area. (**B**) Twitching area for PAO1, PAO1ΔPilA, and Pf^–^ and Pf^+^ clinical isolates, with 4 replicates per isolate (mixed-effects model with isolate as random variable). ***P < 0.001 by *t* test. A lower twitching area indicates a lower twitching ability. (**C**) Plaque assays for Pf4 on PAO1ΔPf4ΔPf6 with negative control, on CPA0056 (Pf^–^), and on CPA0053ΔPf, which are representative of results for all clinical isolates. Plaque assays for other Pf^+^ and Pf^–^ clinical isolates tested are shown in Supplemental Figure 5, for a total of 12 clinical isolates.

Pf phages rely on type IV pili on the surface of bacteria to attach to and infect bacterial cells (15, 38). These pili allow *Pa* to twitch and move along a surface (Figure 5A). Isolates from airways of pwCF are known to downregulate or not express type IV pili (48, 56, 57). We selected Pf^–^ and Pf^+^ isolates, including isolates from patients infected with both Pf^–^ and Pf^+^ clinical isolates over time, and asked whether they had maintained functional pili and could be infected in vitro by Pf. We found that both Pf^–^ and Pf^+^ clinical isolates had reduced pili function, as indicated by a smaller twitching radius compared with the laboratory strain PAO1 (Figure 5B; *P* < 0.05 for both, *t* test). Finally, we investigated the susceptibility to Pf infection of clinical isolates compared to PAO1. While Pf does not need to lyse its host during endogenous replication, lysis is commonly observed at high multiplicity of infection (11, 38, 58). In addition to Pf^–^ and Pf^+^ clinical isolates, we tested the susceptibility of ΔPf mutants of clinical isolates to remove any effect on phage susceptibility of existing Pf infections. While Pf formed plaques on PAO1, we did not observe plaques for any clinical isolates tested. These results extended to isolates CPA0013, CPA0052, and CPA0078, which were from clone types CA08 and CA09 that experienced changes in Pf number, suggesting that Pf infections occurred before the loss of susceptibility observed here (Figure 5C and Supplemental Figure 5). Growth inhibition, characterized as a homogeneous decrease in opacity, was observed for some clinical isolates at the highest titers (1 × 10^11^ PFU/mL or above).

## Discussion

Temperate phages are known to contribute to bacterial pathogenesis and to influence human health but their transmission dynamics can be complex. Here, we have investigated the transmission patterns of the temperate Pf phage in a clinical context, specifically in 3 independent cohorts of pwCF. We found that 60%–70% of clinical isolates were infected with Pf and that most of these Pf^+^ isolates were infected with more than one Pf phage, as determined by the presence of different Pf integrases (13).

We found that Pf phage is typically maintained within *Pa* clone types. Within our dataset, the number and type of Pf prophages infecting each clone type was stable for more than 95% of *Pa* clone types within patients, sometimes over decades. Pf phages with the same integrase identified in clinical isolates from the same clone types in the same patients were more genetically similar to each other than to other phages with the same integrase in different clone types, suggesting that the transmission of Pf phages does not commonly occur between *Pa* clone types in patient airways. In contrast to vertical transmission, horizontal transmission between different clone types is limited in pwCF; the majority of clone types did not gain Pf phages during chronic infections, even when these lasted more than 10 years.

Any analysis of the transmission patterns of Pf phages within a clone type, however, was limited by the low rate of mutation of *Pa* during an infection, estimated at 2.6 SNPs/year (49). This is reflected in slow divergence at the bacterial level in pwCF and even slower divergence of phages. Phages within clone types often shared 100% of their DNA, making it impossible to track phage transmission within a clone type. In addition, the presence of well-conserved genes (e.g., *PA0721*) close to genomic areas of high diversity (e.g., coat proteins) in the Pf genome resulted in poor Pf genome reconstruction during genome alignment and limited our ability to establish transmission outside of patients. Our work established the need for methods tailored to Pf phages in order to fully capture the diversity of Pf phage infections and identify coinfections. Future work could combine short-read and long-read sequencing for optimal assembly and identification of prophages and investigate Pf transmission over larger scales.

Additionally, as these isolates were cultured in a clinical microbiology lab, typically 1–2 different-appearing colonies were identified, reported, and subsequently banked. Using sequencing rather than visual appearance, it has been described that pwCF can harbor tens to hundreds of clones within an individual (59, 60). We may be missing some aspects of Pf phage transmission by not evaluating more clone types per culture and per patient; however, we are likely evaluating the dominant clones within each patient.

Despite the low prevalence of Pf transmission between different clone types within an infection (1 patient), more than 50% of patients saw gains or losses of Pf phages over time. The majority of these changes were associated with a change in clone type (64%, 72%, and 100% depending on the cohort), rather than Pf infection of a clone type previously detected in that patient. Among clone types with changes in Pf number, most lost, rather than gained, Pf phages over time. We investigated factors that could affect the absence of transmission of Pf in patients. Previous work shows that Pf is present in the sputum of some pwCF (9, 61), indicating that Pf phages virions are actively produced by *Pa* in clinical infections. Lack of Pf virion production is thus unlikely to explain the near-complete absence of Pf transmission in patients. Pf has been shown to prevent superinfection of *Pa* by additional Pf phages by inhibiting the function of type IV pili, which are used by Pf (and many other phages) as receptors (38). However, the majority of Pf^–^ clone types also did not gain new Pf phages within the airways, suggesting the presence of additional mechanisms preventing superinfection.

The CF airway is a complex environment and it is possible that the spatial separation between different clone types in the lung, arising either from airway structure or properties of the *Pa* biofilm, would extend to their phages. In addition, *Pa* has been found to undergo many adaptations in the CF airway, generally moving to a less virulent phenotype, including downregulation of pili expression and reduced motility (48, 56, 57, 62), both of which could affect the ability of Pf to reach other bacteria and to attach to the type IV pilus to infect *Pa*. Here, we tested twitching ability, as a proxy for type IV pili function (38), and susceptibility to Pf of clinical isolates, including some sampled from pwCF infected with both Pf^+^ and Pf^–^ isolates. We found that all clinical isolates tested had reduced twitching ability and reduced susceptibility to Pf at clinically relevant titers compared with the reference PAO1. While Pf infections can affect both twitching and superinfection by Pf (38), we observed similar patterns in ΔPf mutants and Pf^–^ isolates, indicating that existing Pf infections were not the main drivers of reduced twitching and Pf susceptibility. Overall, these results support the hypothesis that a reduced function of type IV pili, resulting in the decrease in twitching often observed in isolates from established *Pa* infections, may prevent infection or superinfection of *Pa* by Pf phages.

Given the clinical associations of Pf phage with poor outcomes in CF (9), these data indicate that the initial acquisition of *Pa* with a Pf^+^ isolate may carry prognostic value, perhaps indicating a worse prognosis or trajectory if Pf phage is present. The effect of Pf phage on biofilms and interaction with antibiotics (33, 37) could also indicate high likelihood of establishing chronic infection, or low likelihood of successful eradication. Our groups are currently studying these hypotheses in longitudinal cohorts and mechanistic studies in the laboratory. Additionally, past and present infections of bacteria by lysogenic phages such as Pf can affect the susceptibility of these bacteria to other phages (63–65), including lytic phages used in phage therapy. Our results show that bacteria of the same species and infecting the same airway at the same time can have different Pf prophages, which could result in heterogeneous responses to treatment by both antibiotics and lytic phages. This highlights the need for more comprehensive testing of isolates cultured from sputum. Additionally, many lytic *Pa* phages also use the type IV pilus for attachment and could be less efficacious in *Pa* adapted to the CF airway. Future work should investigate whether our findings can be extended to other phages, both those being used in phage therapy and other phages that may be present as prophages within airway infections. Exploring the effect of adaptation to the CF airway on phage susceptibility and of any other mechanisms potentially at play in vivo (e.g., physical separation of colonies, downregulation of surface receptors, superinfective phenotypes) would provide further insight.

In summary, we have examined the transmission patterns of Pf phage in cohorts of pwCF and observed that new Pf^+^ infections are typically caused by new bacterial infections rather than horizontal transmission of Pf from a coinfecting bacterial clone type. Moreover, Pf^+^ and Pf^–^ strains can coexist within patients and the balance of these strains within individuals can change over time. These results cast light on the transmission of virulence-associated phages in pwCF and highlight the need for more comprehensive sampling strategies.

## Methods

### Sex as a biological variable.

Sex was not considered as a biological variable. Sex was not relevant, as this study focused on bacterial phenotype.

### Collection of Pa isolates at the Cystic Fibrosis Center at Stanford (California cohort).

From June 2020 to June 2023, *Pa* isolates from respiratory cultures from individuals with CF were identified and banked with patient consent for biobanking under IRB no. 11197. Sample numbers and patient IDs are available in Supplemental Table 1.

### DNA extraction and sequencing.

This study includes 162 new clinical isolates from 67 patients at the Cystic Fibrosis Center at Stanford. DNA was extracted using the DNeasy Kit (Qiagen, 69504) and sequenced on Illumina NovaSeq (100 bp paired-end and 150 bp paired-end) (66). We also extracted DNA from 12 samples using the Monarch HMW DNA Extraction Kit for validation using long-read sequencing. Long-read sequencing was performed using Nanopore R10.4.1 flow cells.

### Twitching assays.

Twitch motility was assessed as previously reported (29). PAO1, PAO1ΔpilA, or indicated clinical isolates of *Pa* were stab inoculated through a 1.5% agar LB plate to the underlying plastic dish. After incubation for 24 hours, the agar was carefully removed, and the zone of motility on the plastic dish was visualized and measured after staining with 0.05% Coomassie brilliant blue. Twitching area was measured using ImageJ (NIH).

### Plaque assays.

Plaque assays were performed using PAO1, PAO1ΔPf4ΔPf6, or indicated clinical isolates of *Pa* as recipient strains on LB agar plates. These plates were prepared by adding 5 mL of soft top agar media, consisting of tryptone (10 g/L), NaCl (10 g/L), and agar (5 g/L), mixed with 100 μL of the *Pa* recipient strain (OD_600_ 0.4–0.6) per LB agar plate. Plaque assays were performed by serially diluting purified Pf4 in phosphate-buffered saline (PBS) and spotting 10 μL onto the prepared top agar plates. Plaque forming units were quantitated after 18–24 hours of growth at 37°C.

### Sequence acquisition and assembly.

Raw reads for isolates from the Italy patient cohort were downloaded from the Short Read Archives (49). Raw reads from the California and Italy patient cohorts were trimmed using trimmomatic (67) with the following parameters: java -jar /Trimmomatic-0.39/trimmomatic-0.39.jar PE -threads 8 -phred33 “$input1” “$input2” “$output1” “$output2” “$output3” “$output4” ILLUMINACLIPTrimmomatic-0.39/adapters/TruSeq3-PE.fa:2:30:10:2:True LEADING:3 TRAILING:3 MINLEN:36. We used trim_galore for paired reads to remove Nextera adapaters for raw reads from the Illumina NextSeq. Trimmed reads were then assembled with SPAdes using –isolate and --cov-cutoff auto (68). Assembled sequences were acquired directly from Marvig et al. for the patient cohort from Denmark (48).

### Clone type identification.

Isolates were separated into genetically similar clone types according to the methods described in Marvig et al. (48). Briefly, we used the dnadiff command from mummer/4.0.0rc1 to obtain the number of mutations between each pair of isolates (69). Isolates were then assigned to an existing clone type if they had fewer than 10,000 SNPs when compared with other members of that clone type or assigned to a new clone type if they had more than 10,000 SNPs compared with all other isolates.

### Mutation identification in DNA repair genes mutS and mutL.

Raw reads were aligned against reference genes *mutS* (NP_252310.1) and *mutL* (NP_253633.1) using Bowtie2 (70). We used bcftools to call variants and filtered SNPs using the following criteria: ‘QUAL>=50 && DP>=3 && MQ>=25’. Consensus mutant sequences were then translated to identify non-synonymous mutations.

### Pf prophage identification.

A custom database was built for the conserved Pf genes *PA0718*, *PA0719*, *PA0720*, *PA0721*, and *PA0727* using the command makeblastdb from the blast package. We then used blastn to look for the presence of these 5 genes in all the bacterial isolates with options -word_size 28 -evalue 0.005 and -outfmt “6 qseqid sseqid pident length qstart qend sstart send sframe evalue qlen slen qseq.” We calculated the coverage (percentage of gene length matched by blast) for each gene in R. Isolates were labelled as Pf^+^ if the total coverage across the 5 genes averaged to more than 75%. Our results were not sensitive to changes to this threshold between 35% and 85% (Supplemental Figure 2).

The number of Pf phages infecting each isolate was determined using the phage integrase gene *PA0728*, building on the assumption that an integration site can only be used by one phage at a time. We only recorded the presence of a phage if the integrase *PA0728* was found next to the gene *PA0727* (within 2000 bp), along with either the corresponding excisionase or integration sites. Phages were further classified as containing either the isoform A or B of the CoaB protein. The 5 different integrase, 3 different excisionase, and 2 different CoaB proteins described by Fiedoruck et al. were identified using blastx, based on amino acid sequences (13). We looked for the presence of the 5 different integration sites using blastn with a word size of 6 for all integration sites, except for the Direct Repeat integration site, which was identified using the blastn-short option. Only BLAST hits covering 70% of the sequence with a 70% identity were used for this analysis. This analysis was validated using long-read sequences from 12 different isolates.

### Pf core genome assembly.

When a single phage was found, the boundary of the core genome was defined as the start of the excisionase on one end and the end of the integrase on the other end.

When multiple phages were found, raw reads were aligned to consensus reference genomes corresponding to the type of Pf phages found in that isolate using Bowtie2. We used 10 consensus reference genomes made from phage genomes in Fiedoruk et al. (13), corresponding to all possible combinations of the 2 *PA0723* isoforms and the 5 different *PA0727* integrases. A consensus sequence for each phage in our dataset was then obtained by aligning raw reads to the appropriate reference consensus sequence. Phage sequences from multiple Pf phages with different *PA0723* isoforms cannot be resolved and reconstructed from short reads when present in the same isolate due to highly conserved areas between *PA0723* and the integrase. We omitted these phages from the analysis of Pf pairwise distances presented in this work. This method and these parameters were validated using long-read sequencing data for 12 samples from the California patient cohort.

### Multiple sequence alignment and phylogenetic tree.

Bacterial sequences were aligned and a phylogenetic tree was constructed from the aligned core genomes with Harvest (71). PA7 (BioSample: SAMN02603435) was used as an outgroup. Pf sequences were aligned using Mafft with --adjustdirection (72), and maximum likelihood phylogenetic trees were constructed with FastTree (73). Pairwise distances were calculated using snp-dists.

### Statistics.

All statistical analyses were performed with R. The 2-tailed *t* test and Wilcoxon’s rank-sum test were used to compare the means and medians of normal and non-normal distributions, respectively. The χ^2^ test was used to compare differences in proportions between 2 groups. We used a linear mixed-effect model to compare the twitching area between PAO1 and Pf^–^ and Pf^+^ clinical isolates, with isolate as the random effect, in order to account for both technical and biological variation. Bonferroni’s correction for multiple comparisons was applied. A *P* value of less than 0.05 was considered significant.

### Study approval.

*Pa* isolates from respiratory cultures from individuals with CF were identified and banked with patient consent for biobanking under IRB no. 11197 approved by the Research Compliance Office at Stanford University, Palo Alto, California, USA.

### Data availability.

Sample names and patient IDs are available in Supplemental Table 1. Values for all data points in graphs are reported in the Supporting Data Values file. Raw reads for *Pa* isolates from the California cohort can be accessed through NCBI SRA under BioProject accession PRJNA1188603.

## Author contributions

JDP, PLB, and EBB designed the research study. CM and EBB acquired samples. AG, AK, HAM, PSP, and EBB performed experiments. JDP, NLH, AKS, PF, RJ, and THC analyzed the data. PRS and GADL contributed to the design of the analysis and the writing of the manuscript. All authors contributed to the writing of the manuscript and the design of the figures.

## Supplementary Material

Supplemental data

Supplemental table 1

Supporting data values

## Version 1 04/22/2025

In-Press Preview

## Version 2 06/09/2025

Electronic publication
