# Supplementary material for: New Pseudomonas infections drive Pf phage transmission in CF airways
Source: JCI Insight. 2025 Apr 22;10(11):e188146. doi: 10.1172/jci.insight.188146 (PMC12220970; doi:10.1172/jci.insight.188146)
Supplement: Supplemental data [file jciinsight-10-188146-s161.pdf]

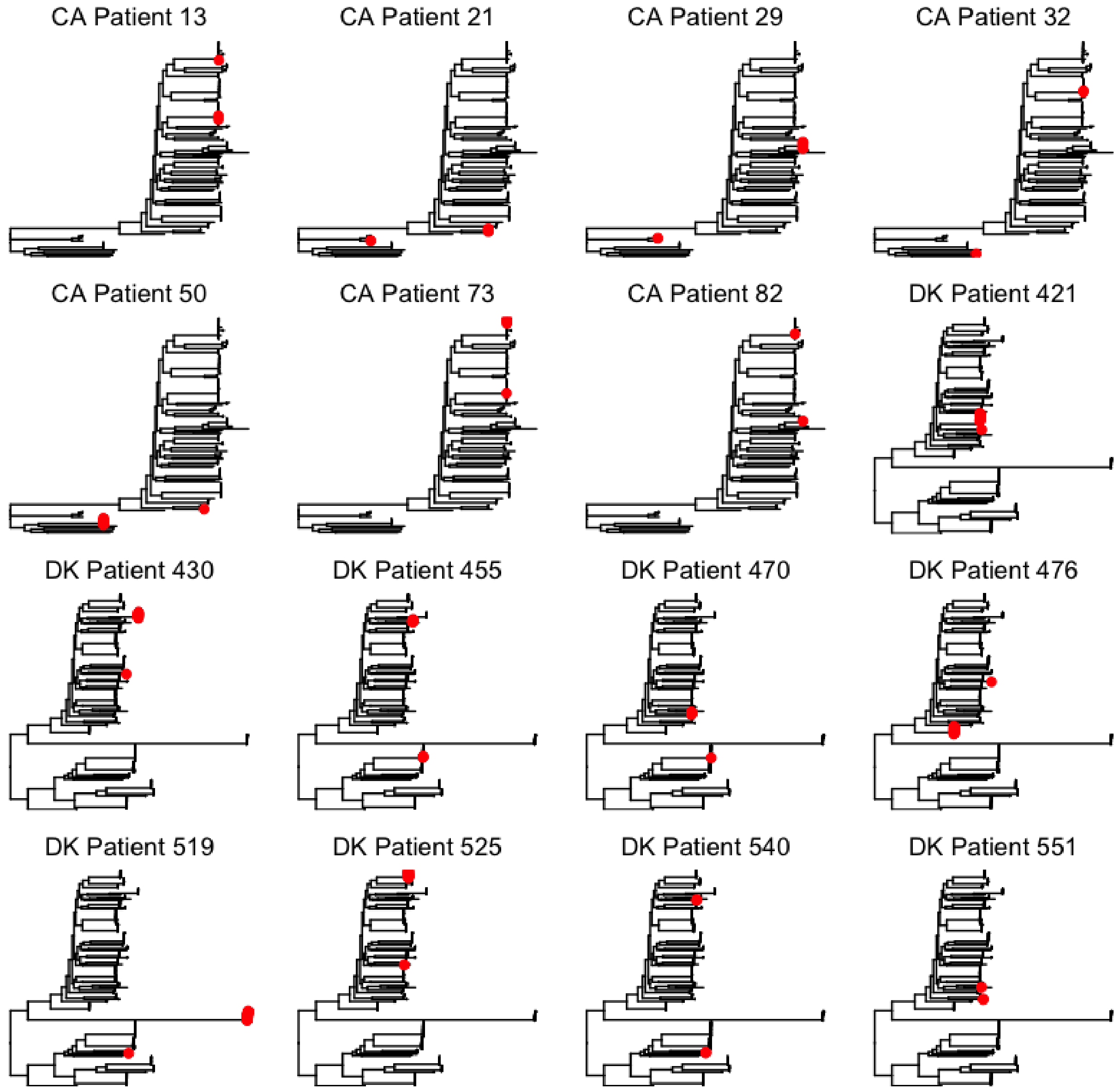

Supplemental Figure 1. Infections of patients by different *Pa* clone types represent de novo infection events. Phylogenetic trees show all samples for that patient cohort. Samples from each patient are highlighted with red circles on the phylogenetic trees. Only patients with isolates from two different clone types are shown here but are representative of patients with more than two clone types.

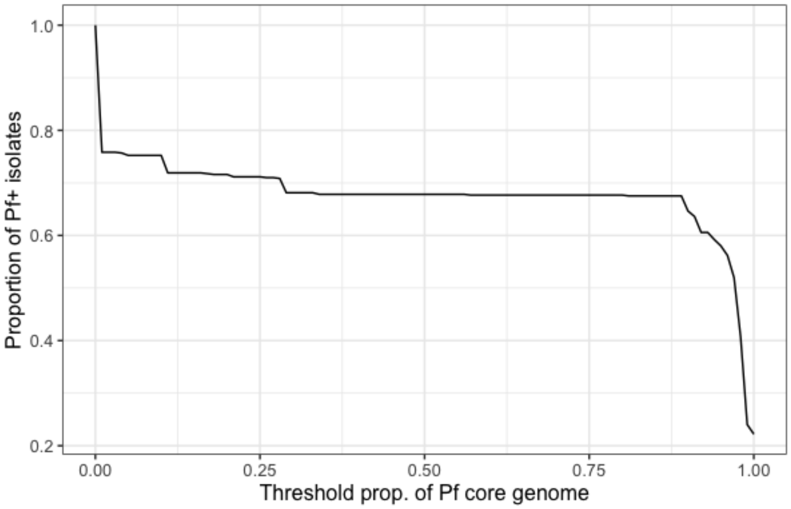

Supplemental Figure 2. Sensitivity of the proportion of Pf+ isolates to core Pf genome threshold required for Pf identification. The core Pf genes considered are *PA0718*, *PA0719*, *PA0720*, *PA0721* and *PA0727*. This analysis included all samples across all three clinical cohorts.

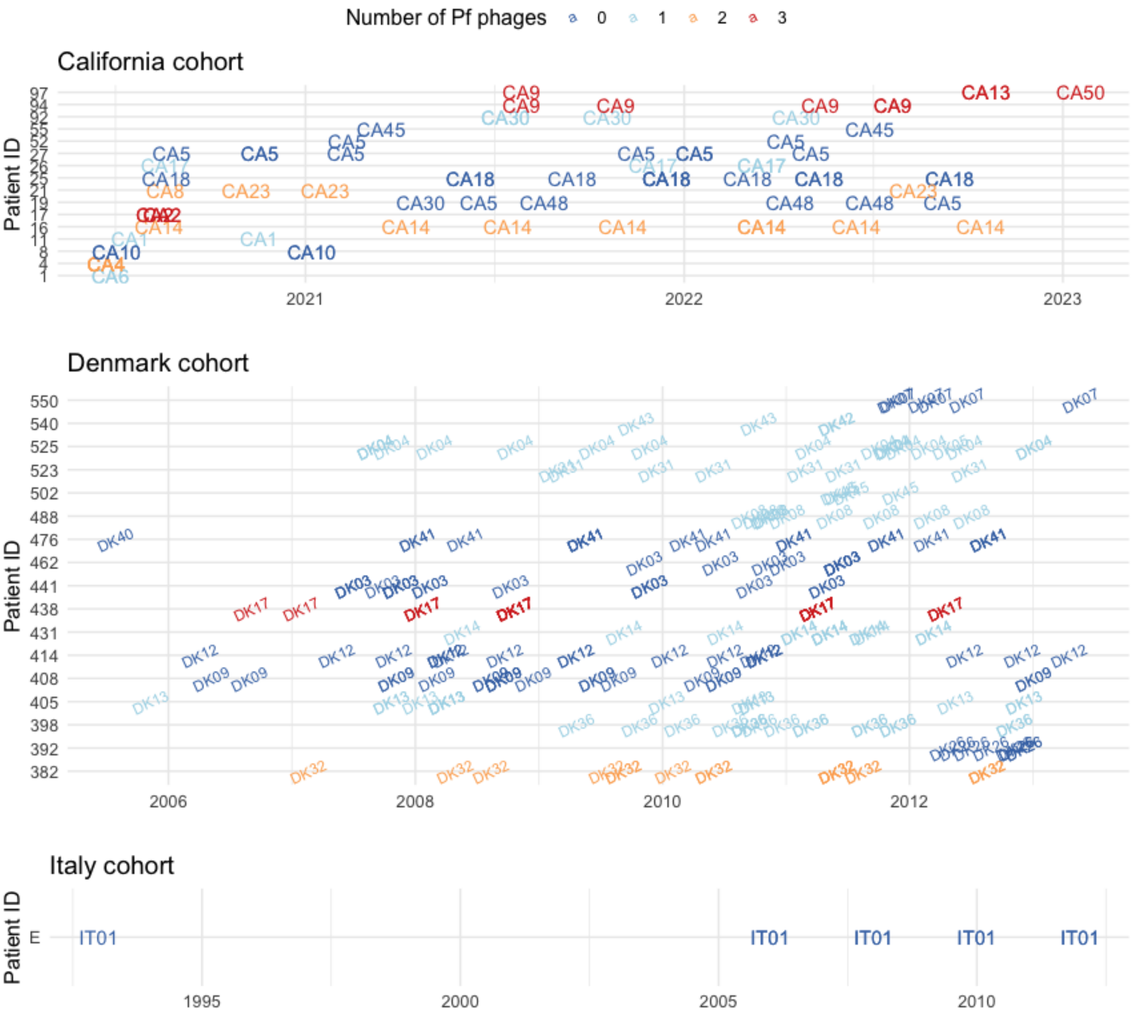

Supplemental Figure 3. Time series of patient samples for patients with isolates with the same number of Pf phages over time. Labels indicate the clone type of each isolate. Colors show the number of Pf prophages in that isolate. Patients with a single sample are not shown.

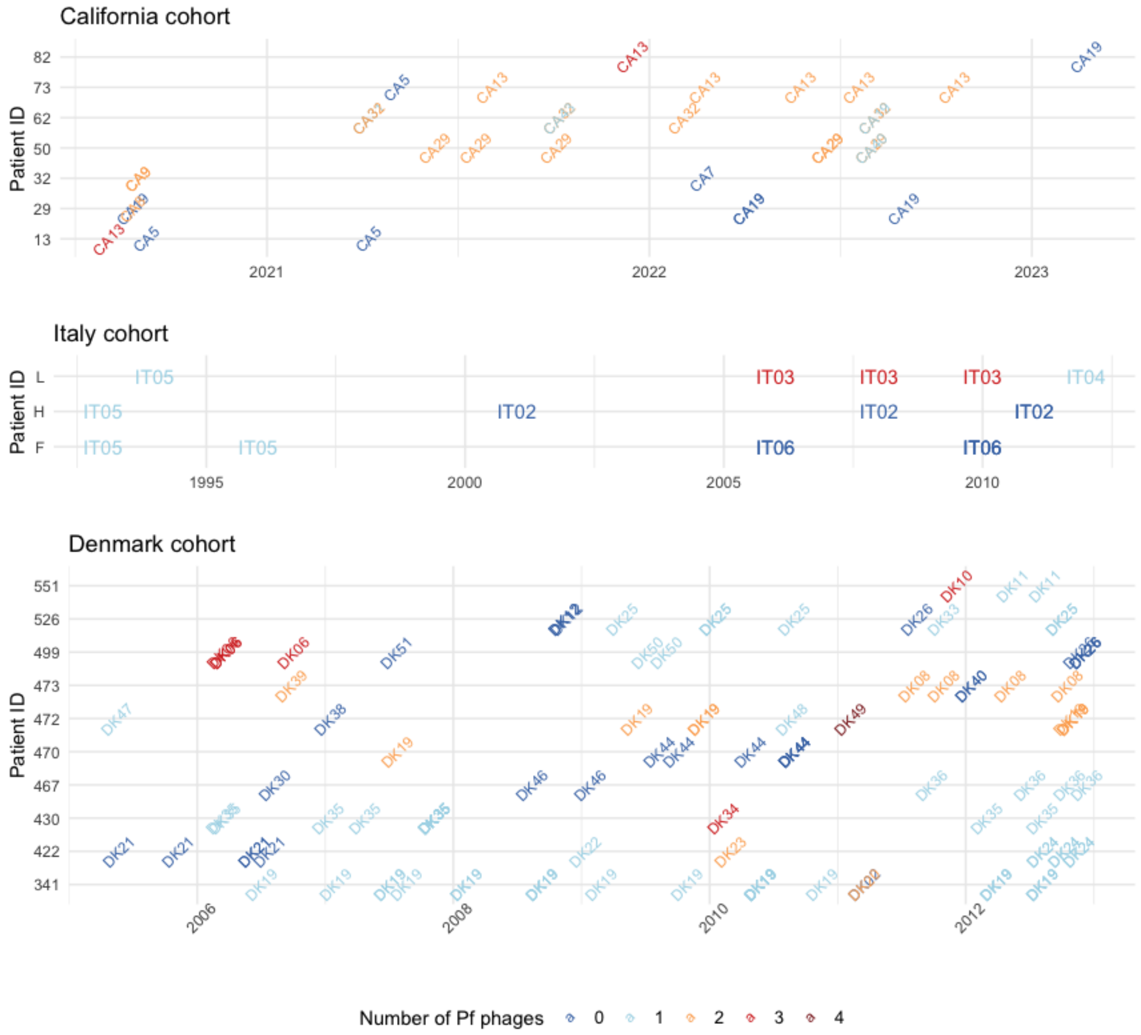

Supplemental Figure 4. Time series of samples for patients with changes in Pf number due to changes in clone types. Labels indicate the clone type of each isolate. Colors show the number of Pf prophages in that isolate. Patients with a single sample are not shown.

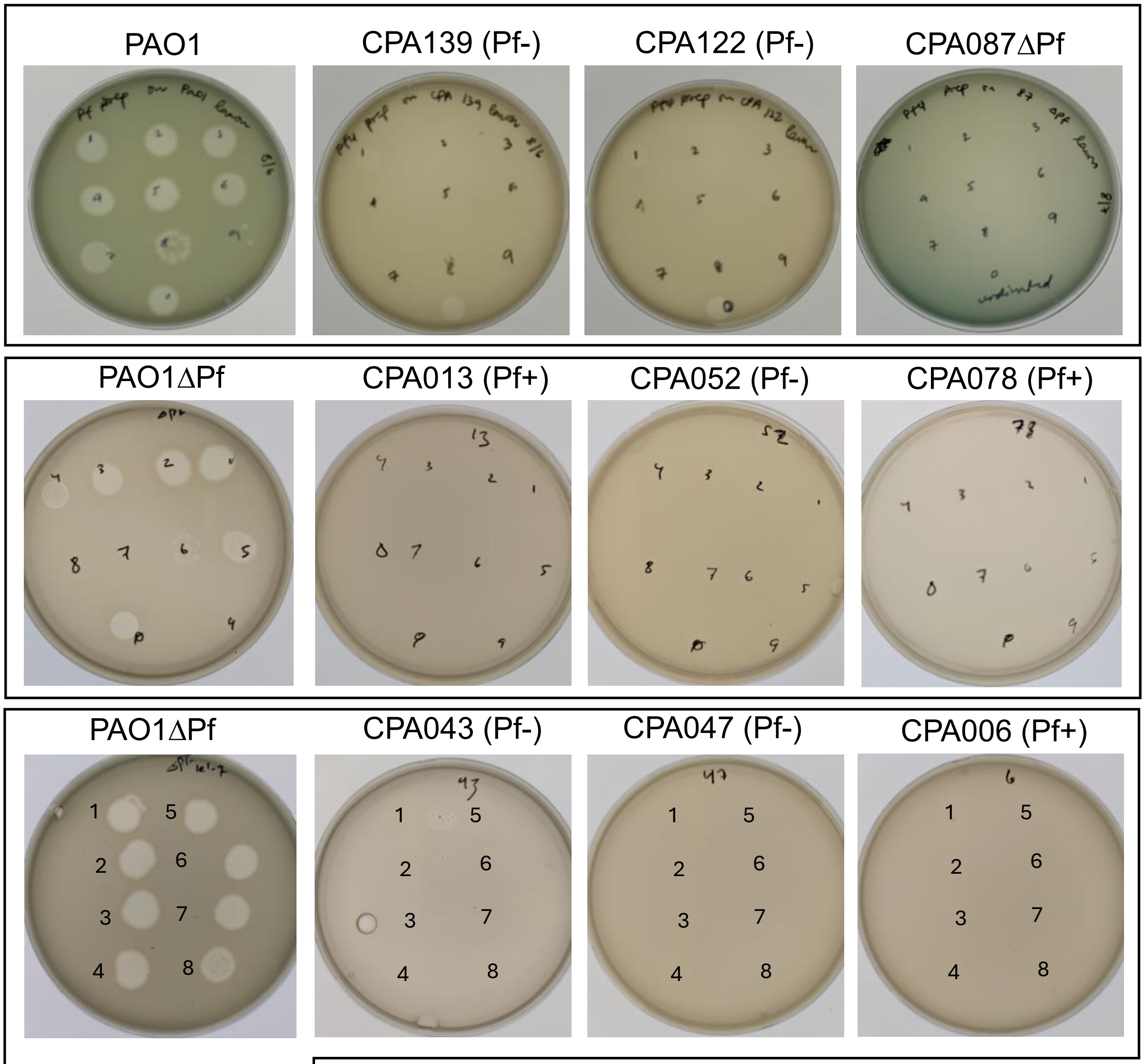

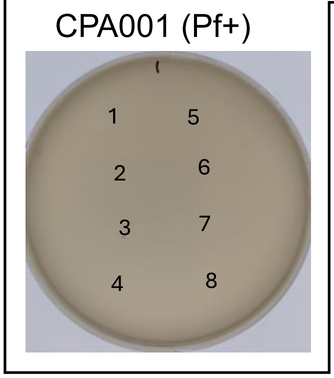

Supplemental Figure 5. Plaque assays for Pf4 phage on PAO1, PAO1ΔPf and clinical isolates. Ten-fold dilutions are given by the number on the plates. Each clinical isolate is grouped with its PAO1 control.
